# Towards an Analytical Biology

**DOI:** 10.2174/0113892029283759231227075715

**Published:** 2024-01-10

**Authors:** Max H. Garzon, Fredy A. Colorado

**Affiliations:** 1 Department of Computer Science, University of Memphis, 373 Dunn, USA;; 2 Department of Biology, National University of Colombia, Bogotá, Colombia

**Keywords:** Biological taxonomies, universal biomarkers, Gibbs energies landscapes, deep structure of DNA oligonucleotides, a geometric definition of species, robust DNA microarrays, DNA chips

## Abstract

This article draws a perspective on the increasingly unavoidable question of whether steps can be taken in genomics and biology at large to move them more rapidly towards more analytical and deductive biology, akin to similar developments that occurred in other natural sciences, such as physics and chemistry, centuries ago. It provides a summary of recent advances in other relevant sciences in the last 3 decades that are likely to pull it in that direction in the next decade or so, as well as what methods and tools will make it possible.

## 1 INTRODUCTION

Unlike Physics and Chemistry, Biology is one of the more recent natural sciences. However, its subject, *life*, is perhaps a far greater challenge given its complexity (even just on earth) and the daunting questions it must address: What is life exactly? Where did it come from? How does it become more and more complex? Can we create it ourselves? Should we, even if we could? Naturally, one might argue that methods in biology have not reached the maturity and depth yet to afford reasonable answers to these questions, in contrast to those of physics and chemistry [1]. For example, Newtonian physics, gravitation and relativity theories provide cornerstones to answer questions as to *why* and *how objects* (like planets) *move* in the universe, one of the fundamental physical questions. Likewise, the atomic theory of matter, the periodic table and analytical chemistry provide answers to fundamental questions in chemistry about the composition of objects and transformations of matter in the real world. In both cases, they undoubtedly made fundamental advances in our understanding of these questions and have allowed us to make fairly accurate predictions about the physical world, moving these sciences along the dimensions of mathematical models and, hence, deductive and predictive power that they envy in the Euclidean geometry and mathematics discovered/invented 2,300 years earlier. Biology, on the other hand, has remained, for the larger part, *an observational and experimental* science, perhaps due to the vast diversity and complexity of living beings and their interactions with their physical environments. For example, in *evolution*, a major branch of biology, the predominant view still deems the changes in living organisms from adult to offspring to be essentially random, not unlike the motion of celestial bodies in the sky just 500 years ago. Is biology so complex that it will be inevitably bound to remain so? Or is it possible to develop, say, mathematically valid models and theories of biological phenomena that bear a sense of universality and hence predictability (akin to the laws of universal gravitation or the periodic table) that would afford it some predictive power about biological phenomena? The goal of this article is to draw a perspective on the increasingly unavoidable question of whether such steps can be taken in and for Biology, what their consequences might be and what might be possible paths to move more rapidly towards these goals. It aims to foster thought and discussion among biologists about directions for biology in the near future rather than facts about what has occurred in the past.

## 2 MATERIALS AND METHODS

The multidimensional complexity (molecular, genetic, morphological, behavioral, ecological) of living beings and their environments is probably the main reason why biology has taken more time in its purpose of predictability with respect to the other sciences. Progress in mathematics, physics and chemistry was not possible until fundamental objects (a physical body like the sun, the moon, or a planet; solid objects, liquids and gases) and concepts (physical position, velocity, acceleration, force; chemical transformations like melting, freezing, annealing) were identified and properly defined. So, what are the fundamental concepts in biology? The obvious and probably correct intuitive answer is that the most fundamental concept in biology is that of *a living organism* or *specimen* (though we cannot define “living,” mind you, just as Euclid could not define “point” or “line” or “plane.”) A seasoned biologist more interested in finding commonalities between organisms aiming to understand life better will probably say that it is the concept of “species.” But *what exactly is a “species”?* The fact is, it appears the answer depends on which biologist group one asks. There are over 20 different definitions, properly documented, as to what might be a reasonable definition of species, each based on particular and relevant aspects in each group [2], which makes it impossible to extrapolate it to others (for example, butterflies cannot be classified under the same parameters used for plants.) Contrary to what one might think, there is no single definition of this term in the 21^st^ century. Yet, a precise definition of species is (even logically) *necessary* to just about anything else in biology. For example, in *taxonomy*, *i.e*., the field responsible for naming and organizing living beings into a comprehensive and understandable whole [3, 4], the concept of species is key. Further, in terms of predictive power, without a clearly defined concept of species, it is *meaningless* to make any prediction about whether this individual belongs to that species or even whether two of them belong to the same species, let alone whether new organisms in a new species will arise on earth with such and such characteristics in the next decade (say, as a result of climate change.) Another fundamental concept is the concept of *reproduction* and *heredity*. One of the first cases of predictability in biology was provided by Mendel and his laws of inheritance [5]. This first baby step led to the current landscape with DNA structure [6] and function, cell organization [7] and the human genome [8]. These advances generate lots and lots of observations and data (the omics), which only compound the problems of a science that is expected to provide information and knowledge to explain and understand the data and the phenomenon of life behind it. They make one feel like Tycho Brahe, in his observatory holding 50 years full of observations of celestial bodies, wishing for a “lazy” graduate assistant (like Johannes Kepler) to synthesize them into three succinct laws of planetary motion. The field of *genomics* has emerged as the subarea that may provide some answers to the questions posed above. For example, *is a concept of a universal biomarker possible* that may be used with *all* species and provide a common frame of reference to enable comparisons across species (or even genera, phyla and kingdoms)? Cartesian geometry was just as instrumental to Newtonian physics to predict the trajectories of celestial bodies as the telescope was to experimental physics actually to suggest and confirm them. Biology is just as lucky because today, we have powerful microscopes, computers and data science. But they are only tools to enable the development of theories, hypotheses and predictions that can be validated and tested with these tools. Given the fundamental role of self-reproduction as a necessary property of life, progress in genomics is necessary for just any kind of biology, such as the origin/evolution of life. A third fundamental but mostly ignored problem is *morphogenesis* and *phenotyping/phenomics*, probably due to its difficulty and lack of appropriate tools. It is well known that DNA is the fundamental structure holding information necessary to create an organism (*i.e*., its phenotypical corporal/physical expression), subject to environmental conditions in a necessary physical milieu/location in the physical world. Progress in this area directly connecting genomics to chemistry, physics and ecology is required. It will have a deep impact on medicine, health and assessing the influence of the environment on a living organism. Fortunately for biology, there has been enormous progress in the last three decades in areas such as self-assembly (particularly in DNA computing), artificial life, and more formal models of living organisms, mostly in computer science. Coupled with emerging tools such as bioinformatics, machine learning, artificial intelligence and data science, it appears inevitable that progress will be made in terms of affording biology with the ability to transform the problem of big omics data into substantial predictive power by making effective use of the enormous amounts of data generated by biological observations, omics included.

## 3 RESULTS

Taxonomy has made considerable progress since its beginnings in the 1700s, when it was based mainly on morphological features. The advent of powerful observational tools (optical and electronic microscopes, molecular sensors and markers and new data sensing and recording technologies and computational algorithms) has produced very useful chromosomal data and moved taxonomy to a scenario of scientific interest in the last few decades. Among the newest and most modern approaches with the aim of increasing taxonomic prediction and precision by homogenizing the species concept, the following stand out: chromosomal studies [9] in plants [10] and animals [11]; evidence that one could use just as well a segment of the mitochondrial marker *Cytochrome Oxidase 1* (*COI*) as a single molecular marker in animals [12]; and the possibility of integrating data from different fields (integrative taxonomy) [13]. They have led to more refined taxonomies, but progress on the fundamental question of what exactly is a “species” remains elusive. Interesting baby steps have been suggested in [14] based on a more fundamental study of Gibbs energy hybridization landscapes [15] that strikes closer to the heart of fundamental principles in biology and bridges the gap with the chemical basis of biology and physics. These definitions also point towards the possibility that we might be able to define the concepts of species using basic Euclidean geometric concepts such as Voronoi diagrams in high dimensions that may cut the Gordian knot of the problem. As a consequence, and perhaps surprisingly given predominant views in biology, they also enable some predictive power for phenotype structure and morphology, *e.g*., the prediction of phenotypic features (such as apotome pigmentation patterns and areas in the heads of *Simulium* larvae) from DNA sequences alone [16]. Ditto for pathogenicity [17]. Now, that does not mean that every feature would be predicted to any degree of accuracy (features like the color of the head spots in black fly larvae do not seem to be predictable [16]), just like statistical models are very useful but do not give certainties and have limitations.

One of the best-known cases of taxonomic complexity among zoologists is also related to black fly (*Simuliidae*), where the presence of species complexes is recurrent [18]. For this reason, several decades ago, the diversity of these insects was addressed through the studies of their giant (polytene) chromosomes [19]. This approach has proven to be quite useful not only to discover the diversity encoded in morphological traits but to advance understanding of evolutionary patterns, chromosomal rearrangements as raw material for speciation in a group, and even predict the geographical distribution of cytoforms (potentially new species) [19] or the location where the organism was grown [14]. However, this cytogenetic work turns out to be costly, requiring intensive training to develop the ability in a human to recognize the bands and universal markers of chromosomes through the microscope [18]. On the other hand, a new thread of research that brings data science to bear on these problems has demonstrated that a methodology based on deep analysis of Gibbs energy landscapes of DNA hybridization of oligonucleotides is possible [15] and may *afford universal biomarkers for genomics, phylogenetic and morphogenetic analyses*, thus contributing enormous savings in time and effort.

These developments led to creative methodological designs, careful records of results and technological development based on machine learning [19]. Currently, multivariate analyses make it possible to predict, for example, the distribution of species on the planet [20, 21] or their extinction [22], as well as the rate of global warming and its effects in the upcoming decades [23], among others. To be useful, such models and theories have to make predictions consistent with the phenomenon of life as it appears to us in a verifiable and reproducible way. But these results arise from a diversity of approaches. They can only be verified *a posteriori*, not with the certainty and inevitability that a physicist or chemist might expect in their fields in their deductive sciences, even if it is about the inherent uncertainty in quantum phenomena (*e.g*., the Heisenberg principle.) Despite these remarkable achievements in the understanding of the nature, organization and workings of the complex world of living beings and their un/predictability, there is still a long way to go in this necessary endeavour.

## CONCLUSION

Very significant progress in the last three decades in a number of areas (sensors, data collection recording and processing technology, computer science, bioinformatics, machine learning, data science, and artificial life, among others), including fundamental advances in hybridization models for genomic analyses, point to a resounding positive answer to the major goal posed in the introduction. It appears very likely *now* that biology is poised to undergo a fundamental transition into an analytic science that necessitate it (*e.g*., by gene editing technologies such as CRISPR [24] and its implications on agriculture and human health) and will make possible the development of universal theories and frameworks for fundamental concepts in biology (such as species, taxonomy, morphogenetics and evolution) that will give it an awesome predictive power, including the limits of such power. These developments will have profound implications and possibly advances for medicine, health and our understanding of life hitherto only dreamed of. And yet, it seems that very deep questions in biology (*e.g*., What is life? How did/does it originate? Where will it lead?) are likely to trouble us forever in science, as pointed out by Urdu poets [25].

## ABBREVIATION

COI: Cytochrome Oxidase 1
